# Biomedical application of 2D nanomaterials in neuroscience

**DOI:** 10.1186/s12951-023-01920-4

**Published:** 2023-06-07

**Authors:** Kangchen Li, Qianting Ji, Huanwei Liang, Zixuan Hua, Xinyi Hang, Linghui Zeng, Haijun Han

**Affiliations:** School of Medicine, Institute of Brain and Cognitive Science, Key Laboratory of Novel Targets and Drug Study for Neural Repair of Zhejiang Province, School of Medicine, Hangzhou City University, Hangzhou, 310015 Zhejiang China

**Keywords:** 2D nanomaterials, Artificial synaptic, Neurological disorders, Glioma, Diagnosis and treatment

## Abstract

Two-dimensional (2D) nanomaterials, such as graphene, black phosphorus and transition metal dichalcogenides, have attracted increasing attention in biology and biomedicine. Their high mechanical stiffness, excellent electrical conductivity, optical transparency, and biocompatibility have led to rapid advances. Neuroscience is a complex field with many challenges, such as nervous system is difficult to repair and regenerate, as well as the early diagnosis and treatment of neurological diseases are also challenged. This review mainly focuses on the application of 2D nanomaterials in neuroscience. Firstly, we introduced various types of 2D nanomaterials. Secondly, due to the repairment and regeneration of nerve is an important problem in the field of neuroscience, we summarized the studies of 2D nanomaterials applied in neural repairment and regeneration based on their unique physicochemical properties and excellent biocompatibility. We also discussed the potential of 2D nanomaterial-based synaptic devices to mimic connections among neurons in the human brain due to their low-power switching capabilities and high mobility of charge carriers. In addition, we also reviewed the potential clinical application of various 2D nanomaterials in diagnosing and treating neurodegenerative diseases, neurological system disorders, as well as glioma. Finally, we discussed the challenge and future directions of 2D nanomaterials in neuroscience.

**Graphical Abstract:**

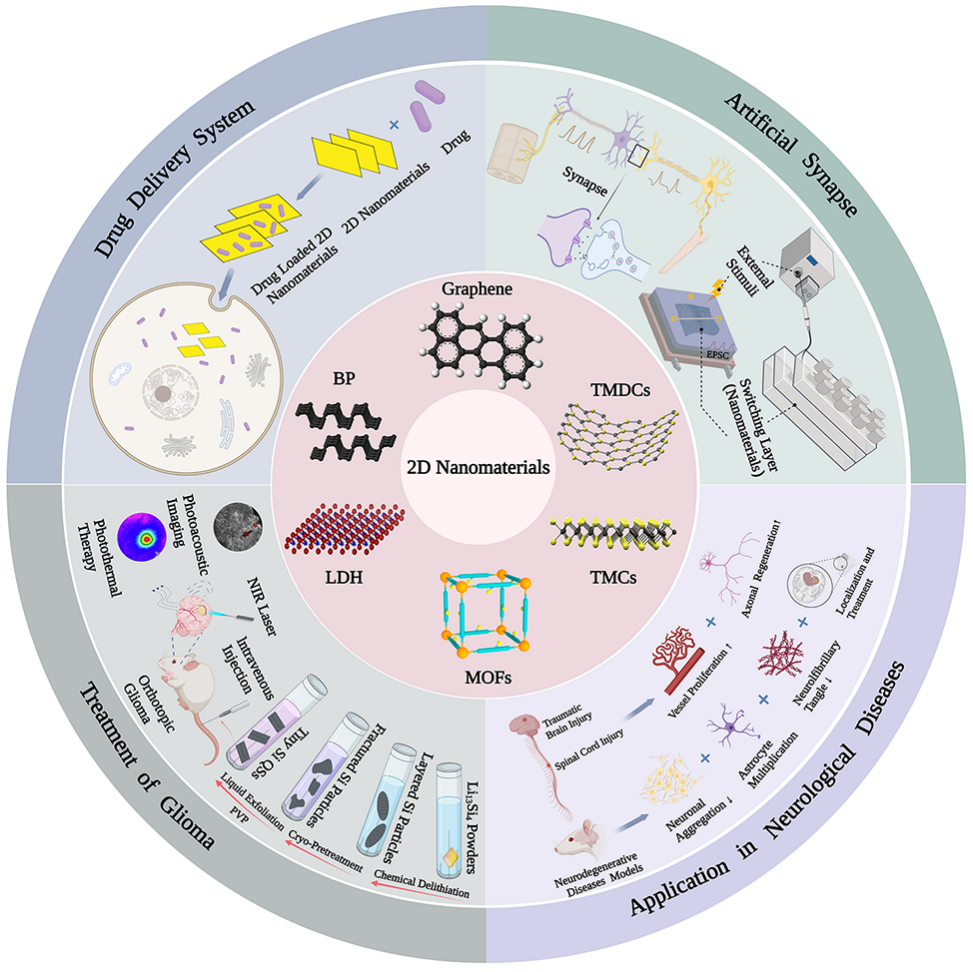

## Introduction

Recently, nanomaterial-based approaches opened up the new potential strategies in the field of neuroscience [1, 2]. Among biomaterials, research on two-dimensional (2D) nanomaterials has been of great attraction. At present, many types of 2D nanomaterials with different structures and physicochemical properties have been developed, such as graphene, black phosphorous (BP), layered double hydroxides (LDHs), transition metal dichalcogenides (TMDCs), and transition metal carbides (TMCs) [3]. Moreover, many 2D nanomaterials have been applied for synaptic modulation, neuroinflammatory reduction, stem cell fate regulation, and injured neural cell/tissue repairment [1, 4, 5]. The nervous system is difficult to repair and regenerate, which is vulnerable to be permanently damaged from injury and disease. The 2D nanomaterials have unique physicochemical properties and excellent biocompatibility to modulate proliferation and differentiation of neural stem cells [6–9]. Therefore, the application of 2D nanomaterials opens up new therapeutic approaches for neural repair and regeneration. Because of the unique properties of 2D nanomaterials that different from bulk materials, such as the electronic, optoelectronic, electrochemical and mechanical properties, they also are good materials for artificial synaptic devices. For example, graphene was initially thought to be cytotoxic, but its high biocompatibility is widely used in artificial synaptic devices [10]. BP as a new emerging 2D semiconductor material having a series of advantages, such as the thickness-dependent bandgap, excellent biocompatibility, and high charge-carrier mobility [11, 12]. TMDCs-based field-effect transistors (FETs) have an ultrahigh Ion/Ioff ratio and good mobility, which can be assembled into low-power electronics. Recently, 2D nanomaterials also attracted much attention in the fields of detection and targeting neurodegenerative diseases (NDs) relying on their ability to cross the blood-brain barrier (BBB) [1]. In addition, in neurological system diseases, 2D nanomaterials also exert great potential through their high surface area, high affinity, and drug delivery functions, effectively inhibiting the inflammatory response after nervous system trauma, and promoting the repair of nervous system damage [13, 14]. The excellent properties of 2D nanomaterials make them also have great potential in the diagnosis and treatment of glioma [15]. Here, we reviewed the most recent studies on various 2D nanomaterials used in neural repairment and regeneration, artificial synapse, and neurological disorders, hoping it could offer an overall understanding of the application of 2D nanomaterials in neuroscience.

## Overview of 2D nanomaterials

2D nanomaterials are a type of material with a thickness of only a single or few atomic layers, and the electrons to move freely in the other two dimensions outside of nanoscale (< 100 nm). The excellent physicochemical and biological properties have led to their extensive application in the tissue engineering and regenerative medicine field. 2D nanomaterials mainly includes graphene, graphene derivatives, BP, LDHs, TMDCs, and TMCs, which have extensively applied in the tissue engineering and regenerative medicine field [2, 16, 17]. Table 1 summarized the biomedical applications of each material.

**Table 1:** Biomedical applications of 2D nanomaterials

Classification	Biomedical applications	References
Graphene	Bone regeneration	[18, 19]
	Neural/Muscle regeneration	[20–22]
	Drug /gene delivery	[23–25]
	Bioimaging	[26, 27]
	Biosensing	[28, 29]
TMDCs	Drug delivery	[30, 31]
	Photothermal therapy	[32, 33]
	Bioimaging	[34, 35]
	Biosensing	[36, 37]
BP	Drug Delivery	[38, 39]
	3D Printing	[40, 41]
	Bioimaging	[41, 42]
LDHs	Drug/gene/protein Delivery	[16, 43, 44]
	Bioimaging	[45, 46]
	Biosensing	[16, 47]
	Tissue engineering	[46, 48]
	Photoluminescence	[49]
TMCs	Biosensing	[50]
	Bioimaging	[51]
	Theranostics	[50, 52]
	Antibacterial activity	[53]

### Graphene and its derivatives

Graphene is the first discovered 2D nanomaterial with perfect physical and chemical properties, consists of a two-dimensional monolayer of sp^2^-bonded carbon atom covalently in a hexagonal lattice [54]. A variety of functionalized forms of graphene-based nanomaterials (GBNs) have been developed and well-studied, mainly including graphene, graphene oxide (GO), carboxyl graphene, and reduced GO (RGO) [55]. As a result of graphene and its oxidized derivatives combine unique properties such as high electronic and thermal conductivities and mechanical strength [56], it has potential applications in wide fields [57, 58], such as biomedicine [59, 60] and artificial synapses [61]. Although graphene is cytotoxic, the non-toxic, biocompatible and water-dispersible graphene layers produced through chemical functionalization can be highly beneficial for pharmaceutical and biomedical applications [62].

### TMDCs

In recent years, a newly emerged kind of 2D nanomaterial, 2D TMDCs has obtained much attention. TMDCs’s generalization formula is MX_2_, comprised of a transition metal atoms (M) sandwiched between two layers of chalcogen atoms (X). Typically, TMDCs include molybdenum disulfide (MoS_2_), molybdenum diselenide (MoSe_2_), and tungsten disulfide (WS_2_). There is a strong covalent bond between layers of tungsten diselenide, but the van der Waals forces between the layers are weak due to their resemblance with graphene [63]. The band gap energy of 2D TMDCs changes with the constituent elements, and even without surface modification, the properties of graphene alter with the changes in the constituent elements [64]. 2D TMDCs are more widely used than graphene in many fields due to their special structure and photoelectric properties. MoS_2_ as a typical layered 2D TMDC has received much attention due to its unique physicochemical, biological and optical properties [65]. TMDCs also attracted numerous attentions for many kinds of applications, such as drug delivery [30] and artificial synapses [66].

### BP

BP is a type of new 2D nanomaterials discovered on top of graphene. Although bulk BP was first synthesized over 100 years ago, the thin-film BP was not discovered until 2014 [67]. BP is one of the most stable allotropes of phosphorus. At present, the known crystal structure types of BP can be mainly divided into four types: simple cubic, orthogonal, rhombic, and amorphous [68]. The thickness of the synthesized BP was 1–2 nm which is about 1–2 layers, and its pore size was between several nanometers and dozens of nanometers [69]. Although BP is considered as a novel 2D material compared to graphene, it is more stable, and has an inherent and perceptible band gap. Multi–channel dynamic sensing measurements showed that BP had high sensitivity, selectivity, and a fast response ability compared to other 2D sensing material [70–73]. Owing to its excellent properties that are similar to graphene, BP has recently attracted increasing attention for a variety of applications, especially in optoelectronics [74–76] and biomedicine, such as photothermal therapy [77], photodynamic therapy [78], artificial synapses [79], drug delivery, theranostics [77], neural repair and regeneration [8, 80].

### LDHs

Due to the attractive morphological features and tunable physicochemical characteristics, the 2D LDHs have drawn the attention of researchers among various inorganic-based nanobiomaterials [81, 82]. LDHs are composed of a positively charged brucite-like layer containing M(OH)_6_ octahedra and an interlayer gallery containing anions and water molecules [83], which are formed by stacking layers of mixed hydroxides of bivalent and trivalent cations, containing hydrated organic or inorganic anions between the layers. LDHs nanohybrids are formed by the encapsulation of diverse anionic species in the interlayer gallery space, including drugs and anionic supramolecular constituents [84]. LDHs are able to interact with organic molecules through anion exchange and, in this way, the anions in the interlayer space are exchanged for other substances of interest. This exchange is characterized by a phenomenon called intercalation and it has several purposes [85]. More importantly, the high chemical stability, pH-dependent biodegradability, and ease of surface modification make LDHs more suitable for biomedical applications [86].

### TMCs

TMCs and nitrides are a kind of 2D materials which are abbreviated as MXenes [87]. MXenes are composed of TMCs, and either nitrides or carbonitrides. They share a general formula of Mn + 1Xn (n = 1–3), where M is an early transition metal (e.g. Sc, Ti, Zr, Hf, V, Nb, Ta, Cr, Mo), and X is a carbon or nitrogen. MXenes combine the metallic conductivity of transition metal carbides/nitrides with the hydrophilicity of their hydroxyl/oxygen/fluorine terminated surfaces in the presence of both complete metal atomic layers and plentiful surface functional groups [88]. This endows MXenes with excellent conductivity, electromagnetic shielding performance and photothermal conversion performance [89, 90]. These tempting properties make MXenes have a wide range of applications in catalysis [91], batteries [92], sensing [93], neural repair and regeneration [94], and therapy [95].

### Other 2D nanomaterials

Apart from the aforementioned nanomaterials, many other 2D nanomaterials including metal–organic framework (MOF) nanosheets and self-assembled 2D nanomaterials were reported contributing to the field of neural regeneration and repair [96].

2D MOF nanosheets have several advantages, such as high purity, ultrathin layer thickness, well refined surface area, abundant exposed unsaturated metal sites, and adjustable chemical composition compared with their 3D counterpart [97]. The drug-loading capacity of 2D MOF nanosheets make them beneficial to be applied in drug delivery and tissue engineering.

Self-assembled 2D nanomaterials with unique physical and chemical properties providing flexible structures and biofunctions compared to inorganic 2D materials, which promote their applications in neural repair and regeneration [1].

In summary, the compatibility and degradability-assisted toxicity issues of various inorganic materials can be addressed considerably by fabricating hybridized structures with the organic components, small size, and reasonable surface functionalization, these nanomaterials appear to be less harmful and possess excellent biocompatibility. The currently available results support further development of these nanomaterials in clinical studies [55].

## Application of 2D materials in neural repair and synaptic simulation

### 2D nanomaterials in neural repair and regeneration

**Fig. 1:**
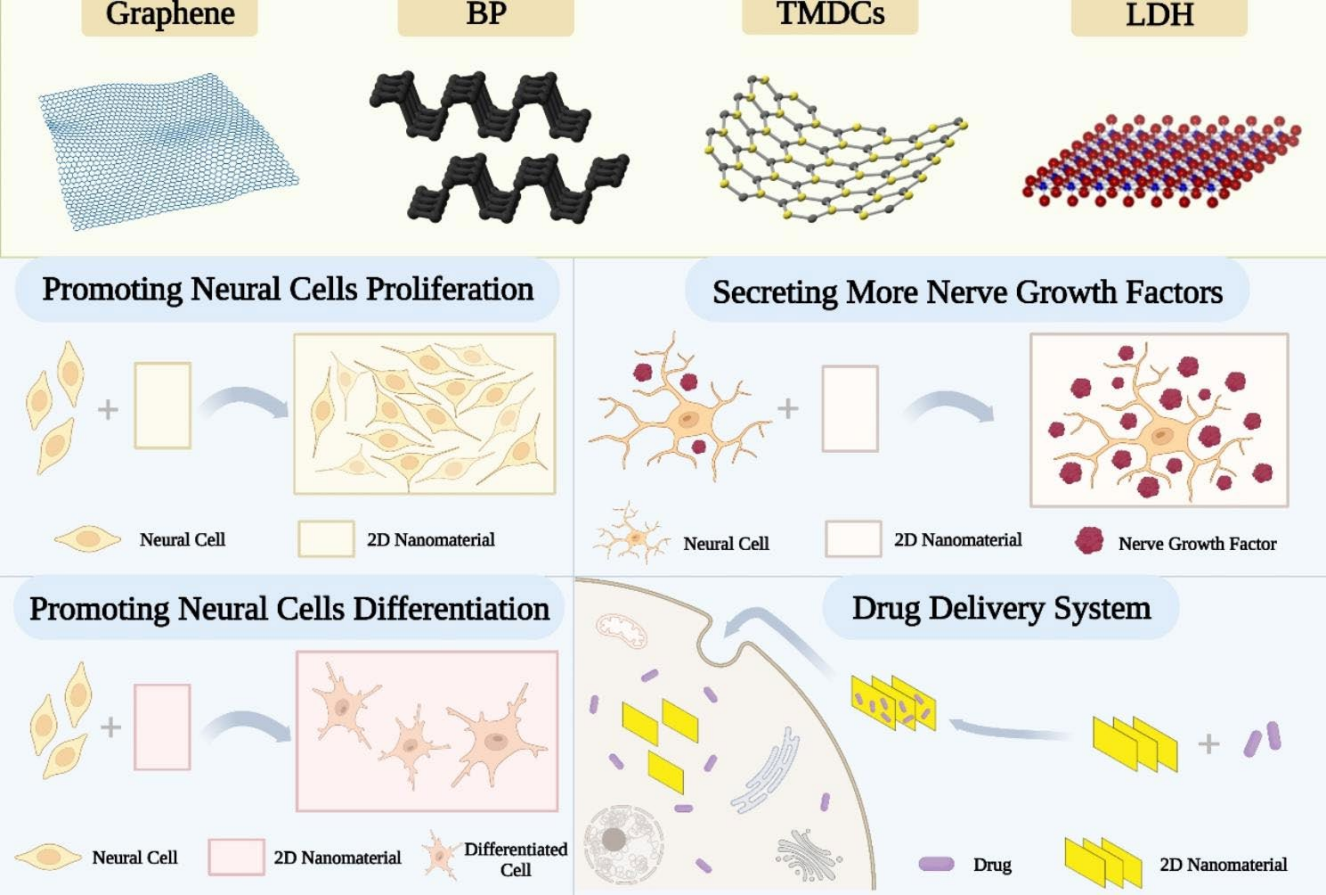
Application of 2D nanomaterials for neural repair and regeneration. The 2D nanomaterials including graphene, BP, TMDCs, LDH, etc. Their roles include promoting nerve cell proliferation and differentiation, stimulating nerve cells to secrete more nerve growth factors, and serving as a drug delivery system to treat NDs.

In the field of neuroscience, it is generally believed that the nervous system has a poor ability to regenerate axonal connections after injury or under disease conditions, and there is currently no effective way to promote the regeneration of nerve cells and the reconstruction of damaged neural pathways [98]. In addition, since the difficulty of drugs reaching nerve damaged areas through the BBB, the design of effective drug delivery systems and implants is also one of the challenges that need to be overcome in the field of neural repair and regeneration. Therefore, the repair and regeneration of the human nervous system is very difficult and is vulnerable to permanent damage due to disease or injury. Recently, the repair and regeneration of nerve has become one of the hot research topics. 2D nanomaterials have unique physical and chemical properties, which can provide more drug loading and good biocompatibility, and have been extensively studied in the field of neural repair and regeneration and have opened up potential therapeutic approaches (Fig. 1).

#### GBNs in the neural repair and regeneration

GBNs are a promising class of 2D nanomaterials. Their excellent electrical conductivity and good biocompatibility make them ideal candidates for neural tissue engineering. Niu et al. revealed that the conductive film composed of graphene and silk fibroin can significantly promote the differentiation of mouse induced pluripotent stem cells (iPSCs) into neurons after iPSCs were inoculated on it. Moreover, the degree of neural differentiation increased with greater quantities of graphene content, and reached the best effect when the graphene content was 4% [7]. There may be a mechanism by which graphene was capable of transmitting electrical stimulation signals and stimulating neuronal differentiation and extension. A recent study found that graphene substrates as cell scaffolds with appropriate electrical stimulation can dramatically increase the number of cells [6]. Supporting cell adhesion and promoting cell proliferation were the main mechanisms by which graphene substrates work [99], showing great potential in regenerative medicine.

**Fig. 2:**
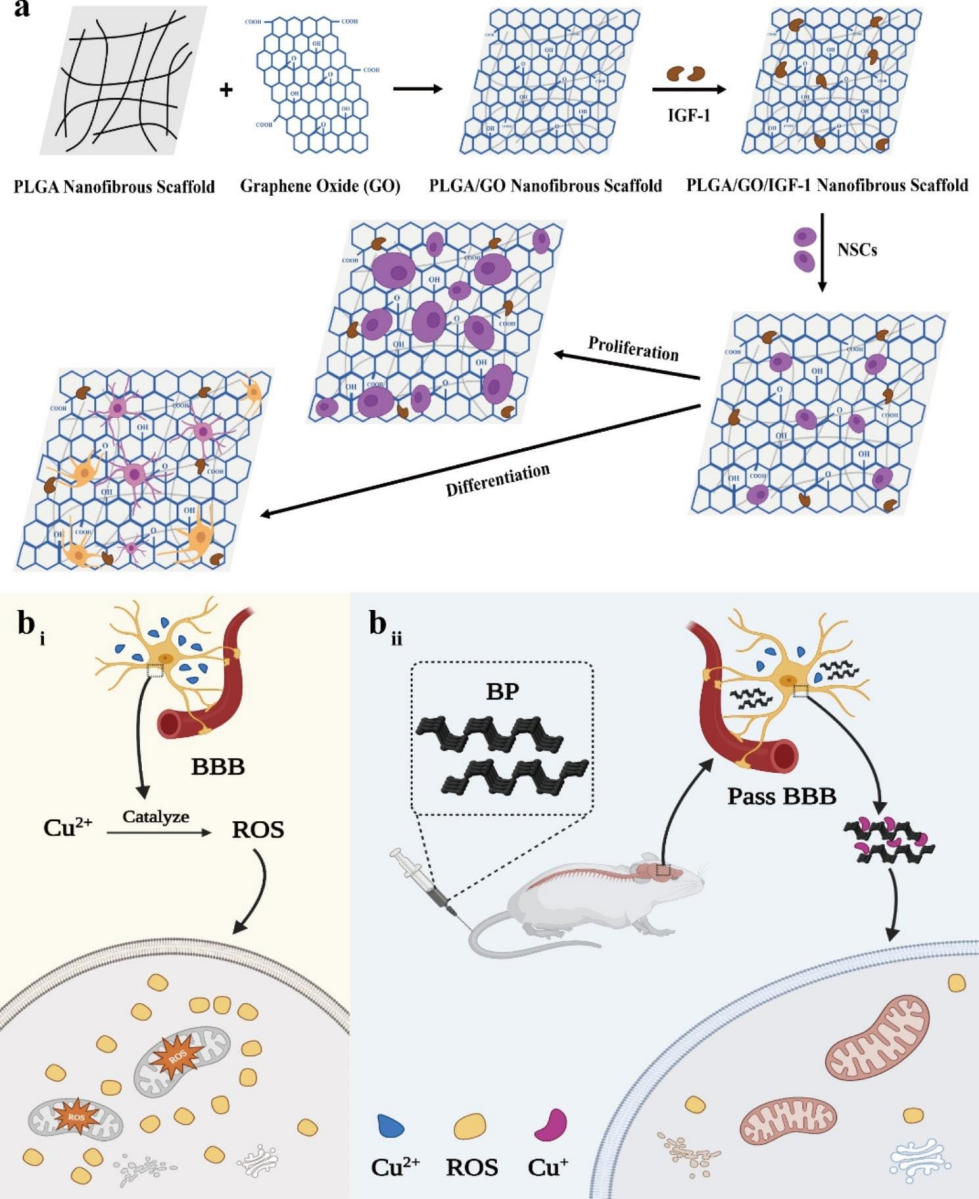
The graphene-based nanomaterials act as nanocomposite carriers for promoting nerve regeneration. **(a)** Schematic illustration of the fabrication of PLGA/GO/IGF-1 nanofibrous scaffold and its application in NSCs culture. PLGA/GO/IGF-1 nanofibrous scaffold significantly increased the proliferation and differentiation of NSCs. **(b)** Schematic illustration of BP nanosheets reduce the generation of ROS by capturing Cu ions for NDs treatment. **(b**_**i**_**)** Cu ions catalyze the production of ROS, which destroys organelles and leads to the occurrence of NDs. **(b**_**ii**_**)** BP passes the BBB and captures Cu^2+^, reducing ROS production and protecting organelles from destruction. Copper on BP nanosheets is mainly in the form of Cu^+^

Additionally, GBNs can act as nanocomposite carriers for promoting nerve regeneration by delivering drugs to the neural cells (Fig. 2a). To cultivate neural progenitor cells (NPCs), Wang and colleagues created functional scaffolds composed of Poly (lactic-co-glycolic acid) (PLGA) nanofiber pads coated with GO and methylene blue (MB) [100]. Although MB has a neuroprotective effect [101] and PLGA is widely used in neural tissue engineering [102], the low compatibility between PLGA and MB prevents them from playing corresponding roles. The result showed that GO coating can greatly strengthen the discharge of MB on the scaffolds, thus improving the ability of NPCs to deal with disease stressors, which provides a possible treatment strategy for NDs [100]. In addition, Qi et al. fixed IGF-1 on PLGA/GO electrospun nanofibers to study the neuroprotective effect of nerve implants. The results indicated that GO greatly enhanced the connection of IGF-1 to the surface of biomaterials, and had the effects of supporting the proliferation and differentiation of neural stem cells (NSCs) [103]. PLGA/GO electrospun nanofibers, as a nanocomposite carrier for immobilizing IGF-1, showed excellent potential in neuroprotection, but its possible mechanism remains to be further studied.

GBNs can also induce the behavior of neural cells. Graphene substrates have been shown to support neural cell adhesion, which in turn promotes cell reproduction [6]. Similarly, Wang et al. found that the number of nerve regeneration factors secreted by neural cells cultured on silk-graphene hybrid hydrogel was significantly increased, and the axon length and proportion of cells also increased slightly [104]. These results indicated that GBNs effectively induce the behavior of neural cells, providing more options for basic and applied research related to neural regeneration.

#### BP in the neural repair and regeneration

BP, a new member of the 2D nanomaterial family, has recently aroused great interest among scientists because of its special physical and chemical properties such as good biodegradability and biocompatibility. Xu et al. designed a double-layer hydrogel platform combined with magnesium-modified BP nanosheets. The experimental results showed that compared with the magnesium-modified double-layer hydrogel platform, it up-regulated the expression of neural-related proteins in NSCs, and significantly promoted the growth of axons in PC12 cells [8]. On this basis, Jing et al. added magnesium-modified BP to methacrylic gelatin to create photosensitive conductive hydrogels, which enabled Schwann cells to secrete more neural growth factors and promote the regeneration of neural fibers [9]. Through these novel strategies, BP is positioned to have a bright future in the field of research on neural repair and regeneration.

The increased level of transition metal ions in cells is a key pathogenic factor in the occurrence and development of many NDs [105]. Phosphorus has the ability to strongly bind to metal ions and can be used as a neuroprotective nano-drug to treat NDs. Chen et al. [106] found that BP nanosheets can cross the BBB, selectively capture Cu^2+^ in the human body, and act as neuroprotectants against Cu^2+^-induced neurotoxicity (Fig. 2b). These exciting results make BP nanosheets a promising therapeutic option for neuro-related diseases.

BP has great potential in drug transportation because of its high specific surface area and non-toxic degradation. Currently, it is mainly used in the treatment of cancer [107, 108], with only a small amount of research on the therapeutic effect of neurological and psychiatric diseases. Jin and colleagues [109] designed a BP drug delivery system containing fluoxetine for the treatment of depression. The results indicated that the system can induce the changes of antidepressant-like cells and shorten the treatment time of depression, which may be a rapid and effective antidepressant pathway.

#### Other kinds of 2D nanomaterials in the neural repair and regeneration

TMDCs share many similarities with graphene, making them promising nanomaterials for neural repair and regeneration. Wang et al. [110] synthesized and characterized MoS_2_ thin films (MTF), which significantly stimulated NSC proliferation, and induced NSCs to differentiate into neurons and neuroglial cells. In addition, Sun and his colleagues [94] fabricated MoS_2_@PEG (PEG = polyethylene glycol) as an effective drug carrier of enalapril in the treatment of spinal cord injury (SCI). The results showed that MoS_2_@PEG can protect the surviving motor neurons and promote the recovery of motor function in mice, which provided the possibility for MoS_2_ to be used as a drug carrier in neural-related diseases.

Because of its superior biocompatibility and minimal cytotoxicity in cellular and animal systems, LDH has been extensively applied in the biomedical area. Wu et al. [111] reported that LDH nanoparticles (LDH NPs) increased self-renewal of mouse embryonic stem cells (ESCs) via activating the PI3K/AKT signaling pathway. He et al. [112] conducted further research based on this and confirmed that LDH NPs have the capacity to boost the expression levels of genes and proteins related to the self-renewal of mESC, and the possible mechanism is that LDH NPs maintain the pluripotency of ESCs through the LIFR/PI3K/AKT signal pathway. These studies indicated LDH is an ideal material for neural repair and regeneration.

### 2D nanomaterials in artificial synaptic simulation

In 2013, Bichler et al. [113] introduced a single nanoparticle organic memory field effect transistor (NOMFET) that behaved similarly to memristors and exhibited the primary behavior of biospiking synapses. This device, also known as the synaptic transistor, was used to display associative memory of Pavlovian learning. Since then, attention has been turned to design synaptic devices [5], and artificial synapses and synaptic electronics have been developed.

The 2D structure materials are known as good materials for artificial synaptic devices existing electronic, optoelectronic, electrochemical and mechanical properties that the bulk materials do not have. Artificial synaptic devices are one of the key components in constructing neuromorphic computing systems. Their main function is to simulate various synaptic plasticity of biological synapses, such as excitatory post-synaptic current (EPSC), inhibitory post-synaptic current (IPSC), short-term depression (STD) and paired pulse facilitation (PPF) [114]. These synaptic properties can also be simulated by basic electronic components such as memristors at two-terminal synapses [115] or transistors structures with three or more terminals [116], and their performance features are often closely related to the 2D nanomaterial systems used, three-terminal artificial synapses have the merits of concurrently transmitting signals and learning. Inorganic and organic electronic synapses have mimicked plasticity and learning. Optoelectronic synapses and photonic synapses have the prospective benefits of low electrical energy loss, high bandwidth, and mechanical robustness [115].

**Fig. 3:**
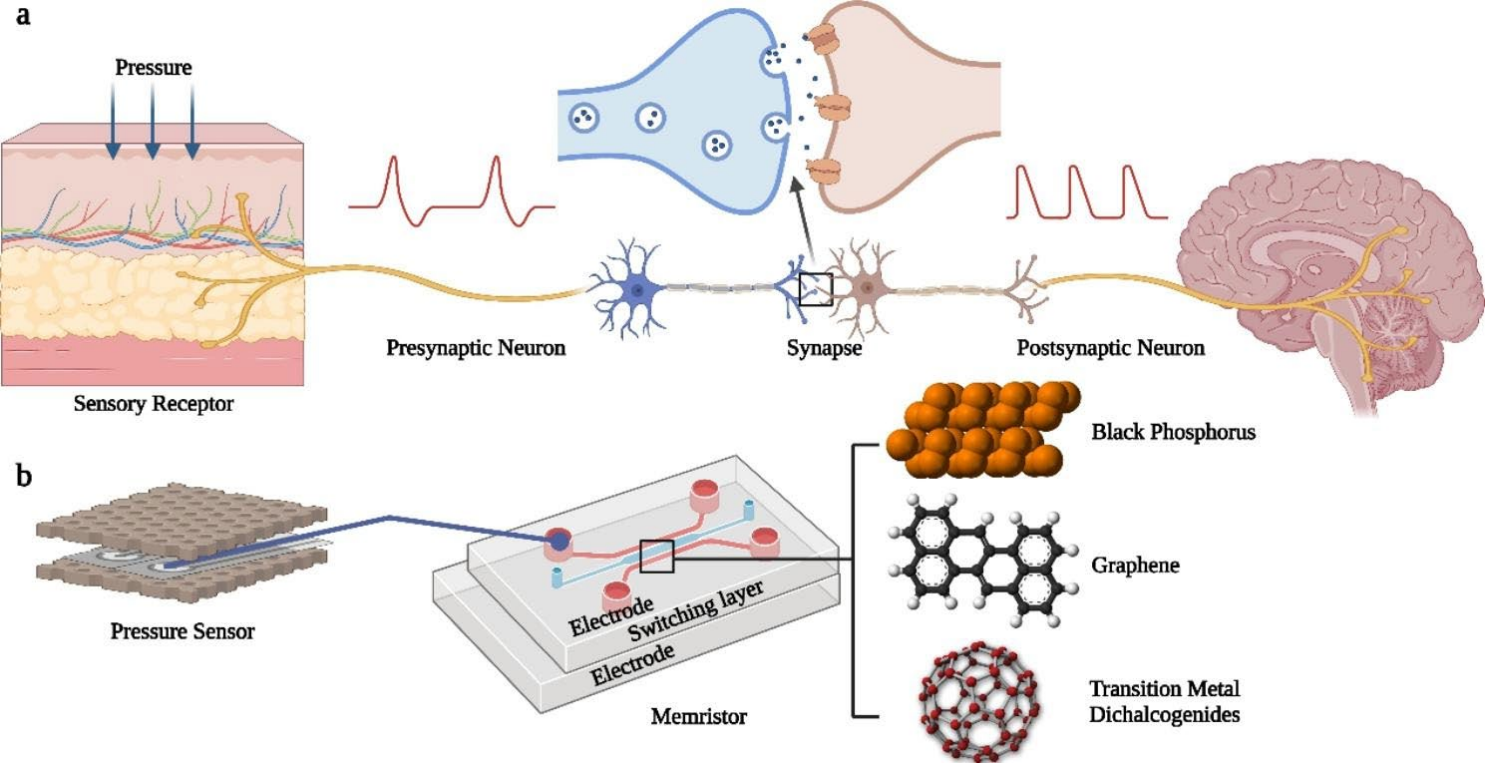
Application of 2D nanomaterials for artificial synaptic simulation. **(a)** A schematic diagram of a biological synapse. **(b)** The artificial synaptic device represents the structure of the memristor. Pressure sensors replace sensory receptors, and memristors based on various nanomaterials simulate various synaptic behaviors. Above and below the memristor are electrode layers, and the middle switching layer can be composed of graphene, BP, TMDCs, etc.

The rising of next-generation computer systems that mimic biological brains has led to an increase in the research of neurobehavior and autonomous learning, which offers a path to neuromorphic engineering [117]. Neuromorphic computer systems that simulate neurons and synaptic transistors is costly because of the lack of suitable materials. In order to further improve the efficiency of neuromorphic computing system construction, the research direction has shifted from equivalent circuits to the simulation of biological synaptic devices. In the process of biological synaptic device research, to conduct a large amount of complex information in different environments and realize high density parallel neural network with low power consumption, it is found that nanomaterials with optical, electrical and chemical properties can be used in artificial synapses [118]. Here, we summarized the applications of 2D nanomaterials in artificial synapses (Fig. 3), including graphene [61, 119], BP [79], and TMDCs [66, 120–122] with ultra-high electrical conductivity.

#### Graphene in artificial synaptic simulation

Although graphene was initially thought to be cytotoxic, it has gained trust in the medical community as a highly biocompatible substrate. Kireev et al. [10] developed a biocompatible bilayer graphene-based artificial synaptic transistors (BLAST) based on the previous large-scale graphene electronic tattoos (GETs), which can mimic synaptic behavior. The BLAST devices leverage a dry ion-selective membrane enabling long-term potentiation (LTP), at least an order of magnitude lower than previous reports on 2D material-based artificial synapses.

Graphene is also combined with other materials to form heterogeneous structures involving in the regulation of artificial synapses. Graphene/Ta2O5/graphene phototransistor exhibits synapse characteristics when visible electromagnetic radiation illuminates the device or positive gate voltage is applied to the device. This device exhibits distinct conductance states modulated by different parameters of incident light, such as the width and number of pulses. The conductance state can be retained for 104 s, indicating LTP, similar to biological synapses [123].

GO was synthesized by the method of liquid phase exfoliation and electrochemical exfoliation on the basis of graphene [124–126]. GO is a promising material with excellent downscaling and compatible with conventional silicon-based devices due to atomically thin and weak van der Waals forces between layers [127]. Furthermore, the band gap of GO can be modulated by controlling the functional groups on the surface, which would allow one to control the electrical properties.

#### BP in artificial synaptic simulation

In comparison to graphene, BP as a new emerging 2D semiconductor material exists a series of advantages, such as the thickness-dependent bandgap, excellent biocompatibility, and high charge-carrier mobility [11, 12], which has been attracted more and more attentions. Yuan et al. [128] reported that the robust and low-power-consumption artificial-synaptic based on BP was successfully manufactured. At the same time, it solved the problem of the instability of exfoliated 2D BP structures. The synapse devices maintain high stability in the air atmosphere and do not show obvious degradation over 3 months. The obtained devices not only implement the main function of synapses, but also perform the function of dendritic neural synapses and simple logical operations, revealing their very strong learning behavior. To utilize a unique photoresponse in BP, few-layer BP flakes were mechanically exfoliated from a bulk single crystal onto the substrates. Monochromatic optical pulses with different wavelengths are used as synaptic stimulation, which optically mimics excitatory and inhibitory action potentials [129].

High-performance and low-power FETs on the basis of integrated circuit field are widely used in the field of synaptic transistors. 2D materials including BP have shown natural advantages for the further reduction in FET sizes due to their natural atomic-level thickness and surface without hanging bonds [130].

#### TMDCs in artificial synaptic simulation

The mobility of the BP FET is much higher than that of the TMDC FET, however, it is not stable and easily decomposed due to the reaction with water and oxygen in the air. TMDCs (e.g. MoS_2_ [131], WS2 [132], WSe2, etc.)-based FETs have an ultrahigh Ion/Ioff ratio and good mobility, which can be assembled into low-power electronics. Krishnaprasad et al. [133] demonstrated ultra-low-variability synapses using chemical vapor deposited (CVD) 2D MoS_2_ as the switching medium with Ti/Au electrodes, which can be used to realize transparent, flexible, ultra-thin memristive synapses for neuromorphic computing.

## Application of 2D nanomaterials in neurological disorders

### Neurodegenerative diseases

Classically, NDs are believed as a group of chronic, progressive diseases characterized by neuronal degeneration and myelin damage, which lead to morbidity and cognitive impairment in older adults [134]. However, there are difficulties in early diagnosis and treatment of NDs, and obstacles in the BBB, and most of the treatments can only temporarily alleviate symptoms [1, 135]. Alzheimer’s disease (AD), Parkinson’s disease (PD), and Huntington’s disease (HD) are three major types of NDs in the nervous system [136], which are associated with protein misfolding or aggregation. The application of 2D nanomaterials in neurodegenerative diseases or neurological system disorders is mainly reflected in early localization diagnosis and subsequent treatment, and its therapeutic effect is exerted by carrying drugs, siRNA and even cells. We will introduce the applications of 2D nanomaterials in these three diseases in detail below and summarized in Table 2.

**Table 2:** Applications of 2D nanomaterials in neurological disorders

Disease	Structure	In vitro /in vivo	Effects	Application	References
Alzheimer’s disease	TPP-MoS2 QDs	In vitro	Mitigate Aβ aggregate-mediated neurotoxicity and eliminate Aβ aggregates	Treatment	[137]
	PEG-LK7@BP	In vitro	Suppress Aβ_42_ conformational transformation from random coil into β-sheet structures	Treatment	[138]
	Dau-GO	In vitro /in vivo	Exert antioxidant and anti-glial aggregation effects	Treatment	[139]
Parkinson’s disease	NiAl LDH/G LBL	In vitro	Track DA from neuronal cell in real-time	Diagnosis	[140]
	MoS2-CPBNPs	-	Potential DA probes	Diagnosis	[141]
	Lf-BP-Pae	In vitro /in vivo	Inhibit neuronal damage; increased the translocation abilities of Lf-BP-Pae across the BBB with near-infrared irradiation	Treatment	[142]
Huntington’s disease	siRNAs-LDH	In vitro	Promote the ability of siRNAs to target and destroy specific mRNAs	Treatment	[143]
	GO	In vitro	Enhance autophagic flow and increase ubiquitination of Htt protein	Treatment	[144]
Traumatic brain injury	GO-pSiNPs	In vitro /in vivo	Gene silence and trauma localization	Treatment	[145]
	CFGO	In vitro /in vivo	Exhibit significant neuro-regeneration potential, promote fast brain regeneration in mice with sham injuries, enhance reactive astrocytes in the hippocampal dentate gyrus after sham injury	Treatment	[146]
Spinal cord injury	PLGA/GO	In vitro	Reduce oxidative stress, promote differentiation of NSCs, and enhance their survival and differentiation	Treatment	[147]
	rGO-MFs	In vivo	Promote axonal and vascular regeneration in injured spinal cords	Treatment	[148]

#### Application of 2D nanomaterials in AD

Defined by the appearance of neuritic plaques and neurofibrillary tangles, AD is a genetic and sporadic neurodegenerative disease that causes an amnestic cognitive impairment in its typical neurological manifestations [149, 150]. Although symptomatic, neuropsychological, and neuroimaging tests are now widely used in clinical, AD still cannot be accurately diagnosed at an early stage [151], and the introduction of clinical testing of 2D nanomaterials may improve the diagnosis of AD. In a recent study conducted by Ren et al. [137], triphenyl-phosphonium bromide-conjugated 1,2-stearoyl-sn-glycerol-3-phosphoethanolamine-N-[amino (polyethylene glycol)-2000]-functionalized MoS_2_ quantum dots (TPP-MoS_2_ QDs) were prepared. MoS_2_ QDs were competent carriers to load TPP across BBB. The composites of MoS_2_ QDs mitigated amyloid beta (Aβ) aggregate-mediated neurotoxicity, eliminated Aβ aggregates, and prevented the expression of neuroinflammation factors such as interleukin-1β (IL-1β), IL-6, and transforming growth factor-β (TGFβ). Mitochondria-targeted enzymes and M1/M2 microglial polarization of MoS_2_ QDs in sera indicated it is a potential candidate to treat AD. In another study, Yang et al. [138] developed an Aβ inhibitor (LK7)-coupled and polyethylene glycol (PEG)-stabilized BP-based nanosystem with high-absorption, large-surface, and conductive. The PEGylated LK7-BP nanosheets (PEG-LK7@BP) are designed to enhance inhibitory potency on Aβ_42_ fibrillogenesis in SH-SY5Y cells [152]. This study showed that the conversion of random coils into sheet structures in Aβ_42_ was suppressed by the presence of PEG-LK7@BP. Further, both of the in vivo and in vitro results of AD models demonstrated that the multi-target therapy of dauricine delivered by GO can protect SH-SY5Y cells from oxidative stress and increase superoxide dismutase levels, as well as relieve cognitive memory deficits and brain glial cells activation in mice [139].

#### Application of 2D nanomaterials in PD

As a result of a pathophysiologic degeneration of dopaminergic neurons in the substantia nigra of the midbrain and the development of neuronal Lewy Bodies, PD is characterized by bradykinesia with rigidity and tremor, with bilateral involvement [153]. Although there are studies proposed PD is probably caused by the degeneration of dopaminergic neurons in the substantial nigra [154], there is no consensus on the pathogenesis of PD at present, and the cause of PD remains unclear. Applying nanomaterials to detect lesions has been regarded as useful tools for the early diagnosis of NDs. In the study by Aziz et al. [140], NiAl LDHs were combined with monolayers of graphene layer by layer (NiAl LDH/G LBL) to track dopamine (DA) from the neuronal cells in real time. This study demonstrated the potential for NiAl LDH/G LBL to serve as a determination window for DA-based PD diagnosis. In addition, prussian blue nanoparticle-supported MoS_2_ nanocomposites (MoS_2_-CPBNPs) are also considered as potential DA probes, but it is lack of supports for the in vitro or in vivo experimental results [141]. The potential application of BP in PD treatment was reported by Xiong et al. [142]. BP containing the brain-targeting ligand lactoferrin and loaded with Paeoniflorin (Lf-BP-Pae) was implanted into SH-SY5Y cells or PD model mice. The treatment results showed that the neuronal damage was inhibited, and near-infrared irradiation increased the translocation abilities of Lf-BP-Pae across the BBB. This study provided a novel approach of drug delivery to treat and prevent PD, as well as other NDs.

#### Application of 2D nanomaterials in HD

Huntington’s disease, also known as chorea, is a hereditary disease with neurodegeneration as a pathological change, which is mainly manifested by involuntary dance-like movements of the upper limbs and head, accompanied by symptoms such as decreased muscle tone [155]. Wong et al. [143] engineered LDH nanoparticles via electrospinning, which had a great ability to deliver small interfering RNAs (siRNAs) through the BBB. LHD scaffolds with siRNAs could promote the ability of siRNAs to target and destroy specific mRNAs, making them particularly useful for treating HD as well as other neurodegenerative conditions. Jin et al. [144] prepared a small, single-layer GO nanomaterial that enhanced the clearance of mutant huntingtin (Htt), the aggregate-prone protein involved in HD pathogenesis. The autophagy induced by GO degraded the expression of LC3-II and Htt, activated class III phosphatidylinositol 3-kinase as well as MEK/ERK1/2 signaling pathways. Furthermore, GO increased Htt’s ubiquitination, which is necessary for Htt’s clearance. GO exhibited a novel biological function in this study and may have implications for the development of neurodegenerative disease therapies based on nanomaterials.

#### Application of 2D nanomaterials in other NDs

There are many types of NDs in addition to the aforementioned, such as a series of motor neuron diseases represented by amyotrophic lateral sclerosis (ALS) that manifest as protein degeneration, with TDP-43 proteinopathies as the characteristic pathological manifestation [134]. Medina et al. encapsulated adapalene within nanoparticles (Adap-NPs) composed of poly(lactic acid)-poly(ethylene glycol) (PLA-PEG). And they finally demonstrated that intravenous administration of Adap-NPs robustly activates retinoid signaling in the CNS, available as a novel nanoparticle platform for the treatment of ALS. The use of nanomaterials improves the efficacy and uses its stealth properties to increase the residence time of adapalene in vivo, thereby exerting the function of a neuroprotector [156]. In addition, calcium phosphate lipids carrying SOD1-ASO [157] and PEGylated polyethyleneimine polymers with a plasmid (pVIVO2) [158] were also involved in ALS treatment by reducing mutant SOD1 levels and gene delivery, respectively. Prion’s disease is another irreversible, progressive neurodegenerative disease, also known as cavernous encephalopathy, with a spongiform-sponge like brain as the main pathological feature [159]. Misfolding in prion proteins leads to β-pleated sheets formation from α-helix, which ultimately results in new abnormal protein termed prion [160]. Binyamin et al. tested that the NE form of pomegranate seed oil (PSO) delayed clinical progression in mice with PrP mutations, a mouse model associated with E200K, indicating that nano-PSO can be effective in the delay of onset of neurodegenerative conditions such as Prion’s disease [161].

### Traumatic diseases of the nervous system

Among the leading causes of morbidity and mortality worldwide of central nervous system (CNS), traumatic brain injury (TBI) and spinal cord injury (SCI) are two major types of traumatic diseases in the nervous system [162]. Below, we will review the recent studies on the applications of 2D nanomaterials in TBI and SCI, and summarized in Table 2.

#### Application of 2D nanomaterials in TBI

TBI refers to an injury to the brain caused by an external mechanical force, which results in a temporary or permanent brain impairment [163]. Joo et al. [145] found that biodegradable mesoporous silicon nanoparticles encapsulated with GO nanosheets (GO-pSiNPs) can directly transport the therapeutic siRNA, and effectively silence the targeted genes in vitro. Neuronal cells in vitro and brain injury in vivo were found to be the most specific targets, and targeting was critical to successful gene silencing, which has great potential to be a therapeutic delivery platform based on RNAi. Pradhan et al. [146] designed an injectable hydrogel containing acetylcholine functionalized GO and polyacrylic acid (CFGO). The well-designed nanomaterial can effectively exhibit significant neuro-regeneration potential, promoting fast brain regeneration in mice with sham injuries, and enhancing reactive astrocytes in the hippocampal dentate gyrus after sham injury.

#### Application of 2D nanomaterials in SCI

SCI refers to damage to the structure and function of the spinal cord due to various causes, resulting in impairment of spinal cord nerve function (such as motor, sensory, sphincter, and autonomic function) below the level of injury [164]. It is a catastrophic injury to the CNS that leads to varying degrees of quadriplegia or paraplegia. Pan et al. [147] designed a PLGA/GO to load IGF-1 and brain-derived neurotrophic factor (BDNF). PLGA/GO-mediated synergistic delivery of IGF-1 and BDNF from the SCI model protected NSCs from oxidative stress, promoting differentiation of NSCs, and enhancing their survival and differentiation. GO is the primary nanomaterial for the treatment of SCI. In the study conducted by Domínguez-Bajo et al. [148], embryonic NPCs were seeded on GO materials in the shape of microfibers (rGO-MFs). Since rGO-MFs had no subacute local toxicity, an experimental rat model of SCI after 4 months of implantation was used to assess their ability to regenerate neural tissue, suggesting that rGO-MF-based scaffolds may promote axonal and vascular regeneration in injured spinal cords. The ability of GO carrying drugs to promote nerve damage repair may be related to the accuracy of graphene providing a better conductivity as well as more accurate drug targets [165].

## Application of 2D nanomaterials in glioma

Glioma is the most common primary craniocerebral tumor caused by malignant transformation of glial cells in the brain and spinal cord, which accounts for approximately 80% of all malignant brain tumors [166]. The diagnosis of glioma requires comprehensive consideration and judgment based on the patient’s medical history, symptoms, signs, auxiliary examinations and postoperative pathology, etc. At present, the most commonly performed examinations include cranial CT and MRI [167]. The treatment of glioma includes surgery, radiotherapy, chemotherapy, targeted therapy and so on. For specific treatment, it is necessary to generally evaluate the functional status of the patient, the expected outcome of treatment, the location of the brain tumor, the degree of malignancy and other factors, so as to formulate an individual treatment plan [168]. Nowadays, the development and utilization of 2D nanomaterials is promising, which is expected to open up a new avenue for the diagnosis and treatment of glioma. The accurate tumor treatment strategy based on 2D nanomaterials has been proved to effectively improve the efficiency of tumor treatment at the level of animal experiments, such as achieving precise targeting of tumors and controlled release of drugs, reducing damage to normal tissues, etc. [169].

### Diagnosis of glioma

**Fig. 4:**
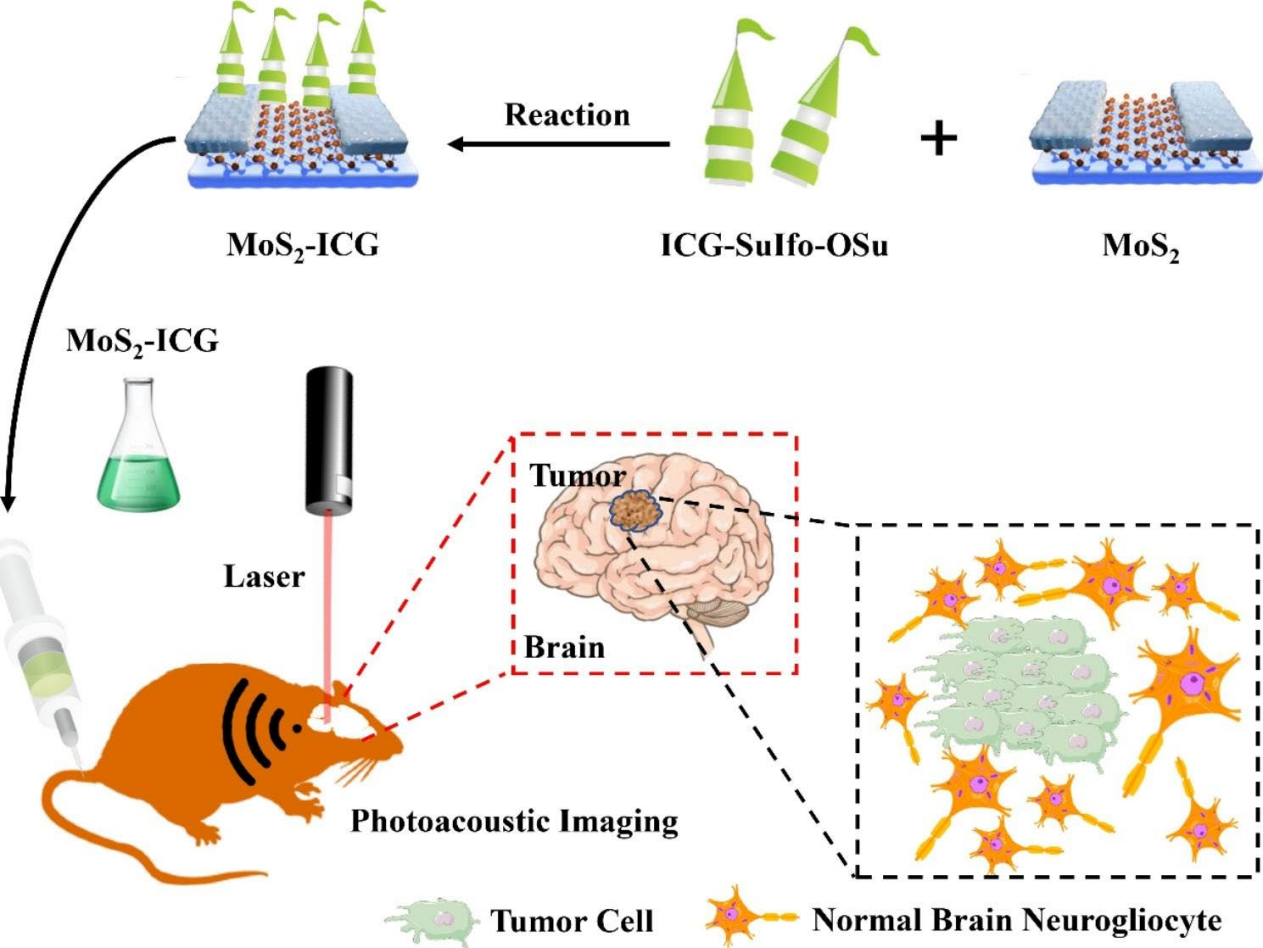
Synthesis and application of MoS_2_-ICG. Using ICG to covalently modify MoS_2_ nanosheets, this covalent connection makes the composite have extremely high optical absorption capacity in a wide near-infrared band, and photoacoustic imaging can achieve lower background noise and greater penetration depth at longer wavelengths. The complex was applied to in vivo photoacoustic imaging of in situ glioma in mice, demonstrating the high sensitivity for visualization of deep glioma.

Photoacoustic imaging is a relatively new molecular imaging technique with strong tissue penetration and high spatial resolution, so it is regarded as an effective diagnostic method for glioma. However, the detection of glioma in deeper layers often requires contrast agents with high photoacoustic imaging sensitivity [170]. Liu et al. [171] reported a MoS_2_ nanosheet composite covalently modified by indocyanine green (ICG), which can significantly improve the sensitivity of photoacoustic imaging [172]. This covalent connection makes it have extremely high light absorption capacity in a wide near-infrared band, which red-shifts the absorption peak of ICG and increases the photothermal/photoacoustic conversion efficiency of ICG (Fig. 4). Using MoS_2_-ICG as a contrast agent, photoacoustic imaging of glioma in vivo at 3.5 mm below the mouse scalp was effectively visualized, and the imaging depth of this process was nearly two times higher than that of previously reported MoS_2_ nanosheet contrast agents [173]. Combined with its excellent biocompatibility and outstanding stability, in translational medicine, MoS_2_-ICG has extensive application prospects for efficient tumor molecular imaging.

### Treatment of glioma

As an important derivative of graphene, a new 2D nanomaterial, GO has aroused widespread interest in the field of biomedicine, and has become one of the research hotspots in nanobiomedicine, especially nano-drug delivering [174]. The main advantages of GO [175] as a nano-drug loading system include: (1) super-large specific surface area, which can achieve ultra-high drug loading rate; (2) strong targeting and easy enrichment in tumor sites; (3) functionalized GO has good biocompatibility and stability under physiological conditions. Shen et al. [176] firstly used PEG modified GO as a protein delivery carrier for cell function regulation. Protein drugs have good biological activity and low toxicity, but they are easily degraded by proteases existing in the organism, and cannot be effectively delivered into cells, resulting in a greatly reduced bioavailability, which seriously affects the efficacy of protein drugs [177]. They also found that GO can not only efficiently load proteins, but also effectively protect them from enzymatic hydrolysis. More importantly, GO can deliver different functional proteins into cells to achieve effective regulation of the physiological functions of cells. On this basis, the researchers covalently modified transferrin on the surface of PEGylated GO, so that the drug delivery system can pass through the BBB, and then target delivery of the anticancer drug doxorubicin (DOX) to the glioma site [178]. In vivo experiments showed that the delivery system could precisely deliver DOX to the glioma site, effectively inhibit tumor growth, and significantly prolong the survival time of tumor-bearing rats.

Photothermal therapy (PTT) uses photothermal conversion to generate heat under laser irradiation, and then causes local high temperature to destroy and kill tumor cells to achieve the purpose of treating cancers [179]. Compared with traditional surgery, PTT, such as radiotherapy and chemotherapy, has the advantages of less side effects and better controllability. Novel 2D nanomaterials such as BP have superior light absorption characteristics and efficient thermal conductivity in the near-infrared region, so they have broad application prospects as photothermal therapeutic agents in the PTT of cancers [180]. Sun et al. [181] prepared ultra-small BP nanoparticles (about 2.6 nm in transverse diameter and about 1.5 nm in thickness) by ultrasonically stripping BP crystals. Studies have confirmed that they have good photothermal anti-tumor effects on glioma. Subsequently, some scholars modified BP nanomaterials with photosensitizers to develop composite nanomaterials that can be used for both photothermal and photodynamic anti-tumor effects. For example, Li et al. [78] developed a BP multifunctional nanoparticle (BPNP) for photothermal/photodynamic synergistic therapy of glioma guided by biological imaging, and confirmed the synergistic effect of PEGylated BPNP on PTT and photodynamic therapy (PDT) through in vitro and in vivo studies. Moreover, BPNP has low toxicity according to histological analysis. Recently, Yang et al. [182] also successfully developed a BP composite nanomaterial capable of dual anti-tumor effects on PTT/PDT. Taking advantage of the high specific surface area of BP nanomaterials, the photosensitizer chlorine6 was modified into BP nanomaterials, so that the prepared BP@PEG/Chlorine6 nanocomposites not only have physiological stability, good biocompatibility and tumor targeting, but also have high photothermal conversion efficiency. However, it has been reported that this therapy still inevitably has a certain degree of tissue toxicity, and the skin damage of mice can be observed in the experiment, but its side effects are still within a controllable range [183].

LDH has a positively charged lamellar structure and an interlayer structure that can exchange anions [184]. The existence of the layered structure helps polar organic compounds to reasonably accommodate between the laminates and form various intercalation compounds. The anions between LDH layers can be easily exchanged with other anionic drugs [185]. Therefore, it has been widely used as a carrier for drug delivery, with the advantages of low toxicity, good biocompatibility, slow and controlled release, targeted delivery, biological imaging and so on [49]. Different drugs and molecules can be loaded into LDH to promote nerve repair and regeneration. Although Curcumin (Cur) has good anti-tumor effect on malignant glioma cells, its biological characteristics limit its application [186]. For Cur delivery, Zhang et al. [187] devised a LDH nanocarrier that showed good targeted therapeutic effect on malignant glioma cells. However, the main disadvantage of Cur is low bioavailability and absorption [186], while the synthesized Cur/LDH nanomaterials can inhibit the invasion and migration of glioblastoma cells to normal tissue cells, indicating that LDH can be applied as a drug delivery vehicle for the treatment of glioma.

Miao et al. [188] prepared two-dimensional silicon quantum sheets (Si-QSs) through a scalable method combining chemical removal and cryo-assisted delithiation, which can be used as high-efficient brain photon nanoparticles for in situ glioma therapy. It has excellent photothermal properties among the reported two-dimensional single element materials [181]. More critically, the low toxicity of Si-QSs maintains a balance between stability and degradability, paving the route for practical clinical transformation considering the storage and action of nanomaterials. After intravenous administration of Si-QSs, the in situ brain tumor were effectively suppressed under the accurate control of photoacoustic imaging, and the survival lifespan of tumor-bearing mice was elevated by two times. Atomic-thin Si-QSs with powerful light-trapping capabilities are potential to offer a stable and valid two-dimensional nanoplatform for the treatment of brain diseases.

## Conclusions and future directions

2D nanomaterials are a new type of biomaterial with enormous potentials, which has great plasticity and unique ability, and they are of great significance to the development of medicine. Because of its excellent biocompatibility and unique physical and chemical properties, 2D nanomaterials have received extensive attention in the field of neural repair and regeneration. 2D nanomaterials have the ability to regulate the neural differentiation of stem cells and the behavior of neural cells. They can also be used as a major component of the drug delivery system to promote the recovery of the injured nervous system. Although 2D nanomaterials offer a promising possibility for neural repair and regeneration, there is still a long way to go before they can be translated into clinical outcomes. First of all, the application of 2D nanomaterials in neural repair and regeneration mainly conducted for GBNs, with relatively less research on other 2D nanomaterials. Therefore, in addition to GBNs, researchers need to continue to explore the related applications of additional 2D nanomaterials. Meanwhile, although the neural repair and regenerative functions of 2D nanomaterials have been supported by lots of studies in animal models [94, 106, 109], their efficacy and safety in the human body, especially in nervous system, are still subject to further debate. Thus, the biosafety of these 2D nanomaterials in the human body should be thoroughly investigated before clinical application.

Successful use of 2D nanomaterials in the fields of brain-inspired computation significantly boosts the possibility of practical use in synaptic electronics [189]. A variety of nanomaterials, including graphene, BP and TMDCs, were utilized to emulate biological functional behaviors. However, synaptic devices with 2D nanomaterials are still far from the ideal computation unit to emulate biological synapses in human brain. Despite enormous efforts, the mechanism of stability and conductivity needs to be further discovered. The size of the artificial synapses should be reduced to enable high integration on chip and the energy consumption of artificial synapses should also be decreased [190].

The treatment application of 2D nanomaterials in neurological diseases has been widely carried out, but these in vitro and in vivo experiments have not been performed in humans, and the progress of combining 2D nanomaterials with imaging is also unsatisfactory. In addition, current research on 2D nanomaterials in mental illness is also limited except for depression [191]. It is still a long way to go before nanomaterials are widely used in the clinical trial of neurological diseases.

2D nanomaterials have the potential to integrate biological imaging detection, drug delivery systems and direct therapeutic reagents, providing a powerful tool for tumor precision medicine[192]. The multifunctional nanotherapy platform based on 2D nanomaterials has become the growing trend of glioma treatment in the future. However, the research of 2D nanomaterials is still in the preliminary stage, and there are still some challenges for its application in human body [17, 193]. Firstly, the toxicity and biological stability of these nanomaterials in the human body environment are still unclear. The current relevant research is in the stage of in vitro and small animal experiments, and experimental studies in large animals are rarely reported. Secondly, the clinical application of nanocarriers will inevitably face with technical and cost issues in the large-scale production of materials. At present, although the preparation technology of nanocarriers in the laboratory is becoming more and more stable and mature, there are still problems to be solved such as complicated preparation steps, high cost, and low efficiency. Thirdly, most ultrasonic and photoacoustic medical devices that assist nanomaterials in tumor diagnosis and treatment are still in the stage of laboratory research. For example, the imaging depth of currently used photoacoustic imaging devices is only tens of millimeters, which is only suitable for research on small animals. Therefore, the innovation of equipment hardware and software to realize clinical application is very important. Although the current research has certain limitations, it is believed that with the continuous development of biomedical engineering and molecular biology, there will be more breakthroughs in the application of novel 2D nanomaterials in neuroscience.

## Data Availability

Not applicable.

## Abbreviations

2D: Two-dimensional
AD: Alzheimer’s disease
ALS: amyotrophic lateral sclerosis
Aβ: amyloid beta
BBB: blood-brain barrier
BLAST: bilayer graphene-based artificial synaptic transistors
BDNF: brain-derived neurotrophic factor
BP: black phosphorus
BPNP: black phosphorus multifunctional nanoparticle
CFGO: cetylcholine functionalized graphene oxide and polyacrylic acid
CNS: central nervous system
Cur: curcumin
CVD: chemical vapor deposited
DA: dopamine
DOX: doxorubicin
EPSC: excitatory post-synaptic current
ESCs: embryonic stem cells
FET: field-effect transistors
GBNs: Graphene-based nanomaterials
GETs: graphene electronic tattoos
GO: graphene oxide
GO-pSiNPs: silicon nanoparticles encapsulated with graphene oxide nanosheets
HD: Huntington’s disease
Htt: huntingtin
ICG: indocyanine green
IGF-1: insulin-like growth factor 1
IL-1β: interleukin-1β
IPSC: inhibitory post-synaptic current
iPSCs: induced pluripotent stem cells
LDH NPs: LDH nanoparticles
LDHs: layered double hydroxides
Lf-BP-Pae: BP containing the brain-targeting ligand lactoferrin and loaded with Paeoniflorin
LTP: long-term potentiation
MB: methylene blue
MOF: metal–organic framework nanosheets
MoS_2_: molybdenum disulfide
MoS_2_-CPBNPs: prussian blue nanoparticle-supported MoS_2_ nanocomposites
MoSe_2_: molybdenum diselenide
MTF: MoS_2_ thin films
NDs: neurodegenerative diseases
NiAl LDH/G LBL: NiAl LDHs were combined with monolayers of graphene layer by layer
NOMFET: nanoparticle organic memory field effect transistor
NPCs: neural progenitor cells
NSCs: neural stem cells
PD: Parkinson’s disease
PDT: photodynamic therapy
PEG: polyethylene glycol
PEG-LK7@BP: PEGylated LK7-BP nanosheets
PLGA: Poly (lactic-co-glycolic acid)
PLGA/GO: graphene oxide-incorporated poly (lactic-co-glycolic acid)
PPF: paired pulse facilitation
PSO: pomegranate seed oil
PTT: Photothermal therapy
RGO: reduced graphene oxide
rGO-MFs: graphene oxide materials in the shape of microfibers
SCI: spinal cord injury
Si QSs: silicon quantum sheets
siRNAs: small interfering RNAs
SLN: solid lipid nanoparticles
STD: short-term depression
TBI: traumatic brain injury
TMCs: transition metal carbides
TMDCs: transition metal dichalcogenides
TPP-MoS_2_ QDs: triphenyl-phosphonium bromide-conjugated 1,2-stearoyl-sn-glycerol-3-phosphoethanolamine-N-[amino(polyethylene glycol)-2000]-functionalized MoS_2_ quantum dots
WS_2_: tungsten disulfide.
